# Assessing Obesity-Related Adipose Tissue Disease (OrAD) to Improve Precision Medicine for Patients Living With Obesity

**DOI:** 10.3389/fendo.2022.860799

**Published:** 2022-04-29

**Authors:** Yair Pincu, Uri Yoel, Yulia Haim, Nataly Makarenkov, Nitzan Maixner, Ruthy Shaco-Levy, Nava Bashan, Dror Dicker, Assaf Rudich

**Affiliations:** ^1^ Department of Clinical Biochemistry and Pharmacology, Faculty of Health Sciences, Ben-Gurion University, Beer-Sheva, Israel; ^2^ Department of Health and Exercise Science, University of Oklahoma, Norman, OK, United States; ^3^ Harold Hamm Diabetes Center, University of Oklahoma Health Sciences Center, Oklahoma City, OK, United States; ^4^ The Endocrinology Service, Soroka University Medical Center, Beer-Sheva, Israel; ^5^ The National Institute of Biotechnology in the Negev, Ben-Gurion University of the Negev, Beer-Sheva, Israel; ^6^ Institute of Pathology, Soroka University Medical Center, Ben-Gurion University of the Negev, Beer-Sheva, Israel; ^7^ Department of Internal Medicine D, Hasharon Hospital, Rabin Medical Center, Petah Tikva, Israel; ^8^ Sackler School of Medicine, Tel Aviv University, Tel-Aviv, Israel

**Keywords:** adipocyte, cell size, inflammation, fibrosis, precision medicine, obesity, adipose tissue

## Abstract

Obesity is a heterogenous condition that affects the life and health of patients to different degrees and in different ways. Yet, most approaches to treat obesity are not currently prescribed, at least in a systematic manner, based on individual obesity sub-phenotypes or specifically-predicted health risks. Adipose tissue is one of the most evidently affected tissues in obesity. The degree of adipose tissue changes – “adiposopathy”, or as we propose to relate to herein as Obesity-related Adipose tissue Disease (OrAD), correspond, at least cross-sectionally, to the extent of obesity-related complications inflicted on an individual patient. This potentially provides an opportunity to better personalize anti-obesity management by utilizing the information that can be retrieved by assessing OrAD. This review article will summarize current knowledge on histopathological OrAD features which, beyond cross-sectional analyses, had been shown to predict future obesity-related endpoints and/or the response to specific anti-obesity interventions. In particular, the review explores adipocyte cell size, adipose tissue inflammation, and fibrosis. Rather than highly-specialized methods, we emphasize standard pathology laboratory approaches to assess OrAD, which are readily-available in most clinical settings. We then discuss how OrAD assessment can be streamlined in the obesity/weight-management clinic. We propose that current studies provide sufficient evidence to inspire concerted efforts to better explore the possibility of predicting obesity related clinical endpoints and response to interventions by histological OrAD assessment, in the quest to improve precision medicine in obesity.

## Introduction

**Figure 1:**
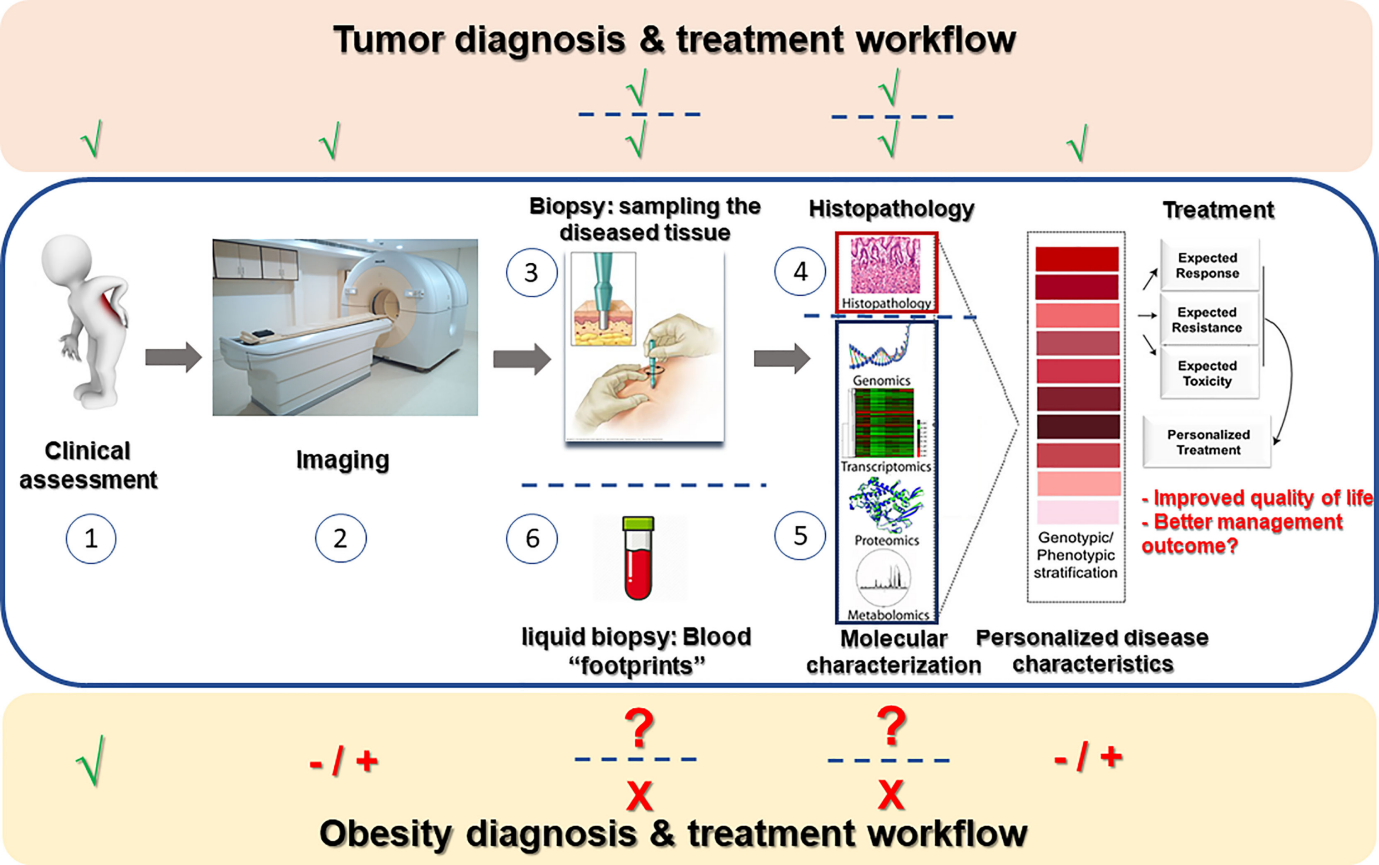
Obesity diagnosis and treatment workflow in the mirror of cancer clinical workup. In a newly-suspected cancer patient, routine workup consists of clinical assessment of the patient (1), various imaging modalities (2) aimed at estimating the extent of tumor invasiveness and spread, sampling of the tumor (3) for microscopic (histopathological) examination (4) and frequently also molecular profiling (5), and nowadays also liquid biopsies (6) attempting to capture free tumor-related molecules (mostly cancer-DNA) secreted by the tumor into the circulation. Data from all above-mentioned procedures is gathered to assemble disease stratification- both anatomical-morphological (grading/staging) and molecular. Based on such stratification, optional treatment approaches can be considered in light of the predicted resistance/response to therapy and estimated severity of toxic effects, and precise disease-appropriate therapy may be determined. Obesity care still awaits a similar personalized approach: While clinical assessment (1) is obviously performed, imaging techniques (2) are rarely performed to estimate the extent/spread of obesity (adipose tissue distribution, adipose tissue thermogenesis, etc.). Adipose tissue sampling (3) is hardly ever performed beyond experimental/research set-ups, and thus histopathological as well as molecular assessment of the diseased tissue is not routinely performed. Overall, obesity workup is deprived of many technologically-available means that could allow to examine if such studies could be used for disease stratification and better personalization of the treatment of people living with obesity.

Recent decades had seen tremendous advances in the ability to personalize treatment to the specific patient based on numerous parameters, resulting in improved care (increased efficacy, less side-effects, etc.). In the treatment of cancer, the diseased tissue – the tumor – is routinely assessed macroscopically, microscopically, and molecularly; advanced imaging technologies are used to determine the extent/spread (or ‘stage’) of the disease, and liquid biopsies – cancer-related mutations that are captured in blood samples and serve as biomarkers or indicators of the tumor driving mutations – are increasingly used. These assist in determining prognosis of the patient and predicting response to different treatments, based on which the most efficacious treatment(s) is being offered, and non-efficacious or possibly harmful treatments avoided. The above personalized/precision care approach hardly reflects the situation in current obesity care (**Figure 1**). Obesity is still formally defined anthropometrically by body mass index (BMI), and although personal characteristics and preferences of the patient are taken into consideration to offer the best possible care, only limited investigations are routinely performed to predict future development of obesity-related complications or the response to different modes of therapy. This is despite the fact that with its enormous prevalence, obesity is clearly not a single entity, and its heterogeneity likely encompasses potentially definable sub-phenotypes that would respond differently to specific treatments. Indeed, debatable entities reflecting obesity sub-phenotypes, such as ‘metabolically-healthy’ versus ‘metabolically-unhealthy’ obesity had been proposed. Yet, largely, these definitions rely on whether the patient had already-developed health implications of obesity rather than attempting to predict their occurrence. Jointly, the clinical resolution at which obesity is currently defined is hardly helpful in optimizing care and intervention options to the individual patient, largely failing to provide prognosis, or predict side-effects or efficacy of available and emerging interventions.

Human adipose tissue is one of the most evidently altered tissues in obesity. Intriguingly, some of these alterations are clearly more evident – in their prevalence and/or extent – in obesity phenotypes with greater metabolic risk, as assessed mainly in cross-sectional studies. Such alterations, sometimes collectively related to as adiposopathy, and which we herein propose to name Obesity-elated Adipose tissue Disease (OrAD), include features such as adipocyte hypertrophy, adipose tissue inflammation and adipose tissue total and pericellular fibrosis. Many of these OrAD features can be assessed using rather basic histopathological examinations, and/or molecularly. Adipose tissue distribution and the extent of ectopic fat accumulation may be viewed as a key feature of OrAD, providing prediction of obesity-related clinical endpoints (1, 2). Indeed, high waist-to-hip ratio – an anthropometric measure of central adiposity and/or limited lower-body fat expandability (3), has been recently shown as a central component of a proposed new definition for impaired metabolic health, also among patients with obesity, and to predict all-cause and cardiovascular mortality (4). Some studies have also attempted to identify molecular patterns that could potentially help personalize obesity care (discussed briefly later) (5, 6). However, this review will focus on histopathological OrAD features that had been shown to predict subsequent clinically-relevant obesity outcomes – risk of developing obesity-related diseases, and response to intervention. We emphasize OrAD features that can be easily assessed and implemented by many centers treating obesity, and provide a practical guide approach to implement them in the obesity clinic routine. We hope to convey the message that adipose tissue retains clinically-relevant information that is readily available, and which is currently under-utilized in the quest to better personalize obesity care.

## Histopathological OrAD Features That can Predict Obesity Outcomes

The following sections will each deal with a specific OrAD histopathological feature for which we found peer-reviewed publications that provide proof-of-principle that they may predict future development of obesity endpoints and/or response to anti-obesity intervention. Available literature on each OrAD feature were divided into 4 categories, i.e., tiers, representing different “levels of linkage” between the specific OrAD feature and obesity related endpoints: Tier 1 includes examples of cross-sectional studies of a single time point, demonstrating association between an OrAD feature and an obesity phenotype. Tier 2 highlights longitudinal studies that describe a correlated *change* between an OrAD feature and a clinical characteristic based on two time-point assessment. (Note: We prioritized studies that demonstrate correlated change between both the OrAD feature and the clinical parameter over studies that merely describe a significant change (delta) in the OrAD parameter per-se). Tier 3 highlights studies describing possible mechanisms for the link between the OrAD feature and clinically-relevant endpoint(s). Tier 4, the focus of this review, are follow-up studies that demonstrate association between *baseline* OrAD feature, and *subsequent* (incident) development of obesity-related endpoint and/or response to intervention. In line with the focus of this review, studies in tiers 1-3 will be mentioned only briefly and summarized in a designated table for each OrAD feature, and tier 4 studies will be discussed in more detail.

### Adipocyte Size

Adipose tissue can expand by recruiting more adipocytes – i.e., hyperplasia, likely from adipocyte progenitor cells present within the tissue, and/or by increasing adipocyte size/volume (hypertrophy) (7–9). Indeed, the adipocyte can uniquely vary in size, ranging from <20 to 300 µm in diameter (10). Cross-sectional studies, even if not unanimously, link larger adipocytes with clinical parameters consistent with greater metabolic risk [**Table 1**, tier 1, and excellently reviewed in (10, 44)]. Yet, some studies questioned whether these cross-sectional associations are superior to, or provide associations independent of, measures of total body mass and/or fat distribution (45–47). These inconsistencies in the literature possibly suggest context-specificity – i.e., differences between ethnicities, sex, BMI range, and the specific fat depot studied [ (11, 44), and further discussed below]. Longitudinal studies demonstrate that decrease in adipocyte size that accompanies weight loss associates with improved obesity-related disease endpoints in some (**Table 1**, tier 2), though not all studies (48, 49), and pioglitazone treatment actually induced improved insulin sensitivity that associated with increase, not decrease, in adipocyte size (50). Mechanistically, several underlying processes were proposed to explain the link between enlarged (hypertrophic) adipocytes and metabolic dysfunction (**Table 1**, tier 3), most notable of which are: i. larger adipocytes have altered metabolic and endocrine functions compared to their smaller counterparts. In particular, they seem to be more insulin resistant, and more lipolytic (35–37). The latter feature results in greater release of non-esterified (free) fatty acids to the circulation, which in turn may contribute to metabolic dysfunction and cardiometabolic risk (51). ii. Large adipocytes are more pro-inflammatory: they secrete pro-inflammatory cytokines and chemokines that support inflammatory cell accumulation (31, 32). In addition, hypertrophied adipocytes tend to die *via* an unclear/non-classical cell-death program (necrosis, apoptosis) (33), which nevertheless seems to be pro-inflammatory, or at least contribute to macrophage recruitment to the tissue. Recently, the link between pro-inflammatory cytokine secretion by larger adipocytes was attributed to a state of premature senescence, and a senescence-associate secretory profile (SASP) (34).

**Table 1:** OrAD feature - Adipocyte size.

		References
**Tier 1**: Cross-sectional association Adipocyte size correlates with obesity-related diseases and/or cardiometabolic risk, *beyond other adiposity measures	- Insulin resistance.	(11–15)
	- Dyslipidemia/particularly visceral adipocyte hypertrophy, in women.	(16, 17)/ (18, 19)
	- A subcutaneous adipocyte size threshold could be identified beyond which association with T2DM increases.	(20)
	- Non-alcoholic fatty liver disease.	(21, 22)
	- Coronary artery disease (epicardial adipocyte size).	(23, 24)
	- Polycystic ovary syndrome.	(25)
**Tier 2**: Longitudinal co-association *Adipocyte size changes during intervention associate with changes in obesity-related phenotype/risk*	- Glucose metabolism and/or insulin sensitivity.	(26–30)
	- Cardiovascular risk.	(29, 30)
		
**Tier 3**: Main proposed mechanisms *for the link between adipocyte size and clinical characteristics.* *(human studies)*	- Larger adipocytes are pro-inflammatory:	
	i. have a more proinflammatory secretome.	(31, 32)
	ii. Greater tendency for adipocyte cell death.	(33)
	iii. Display premature senescence.	(34)
	- Dysregulated lipolysis (more FFA release).	(35–37)
	- Altered adipokine profile.	(14, 31, 32)
**Tier 4**: Prospective/predictive studies	*Incident T2DM*	
*Baseline adipocyte size predicts subsequent progression of obesity-related conditions, and/or intervention outcome*	- Larger SC adipocyte size predicts development of obesity-related T2DM in Pima Indians *(33 cases of 108, 9 years average follow up)*.	(38)
	- Larger abdominal SC adipocytes (less so femoral) predict incident T2DM in women *(36 cases of 234, 25 year follow up)*.	(39)
	*Experimental overfeeding response*	
	- Larger SC adipocyte size predicts *lower* fat mass and hepatic fat gain, and a *smaller* decline in insulin sensitivity *(n=29 men, mostly non-obese; n=31 males and females, BMI 25-35 Kg/m^2^).*	(40, 41)
	*Obesity intervention outcome*	
	- Larger SC adipocyte size predicts *lower* resolution of T2DM+insulin resistance 6 months after bariatric surgery *(2 cohorts; resolution in 61 of 79, and 13 of 33)*.	(20)
	- Smaller “adipocyte density” (indicative of larger omental adipocyte size, which in itself was not significant) predicted greater reduction in carotid intima-media thickness following metabolic surgery *(n=40).*	(42)
	- Hypertrophic SC adipocytes predicted *greater* improvement in insulin sensitivity in response to dietary intervention or bypass surgery *(n=100 and 61, respectively).*	(43)
	- Larger adipocyte morphology value predicted *greater* improvement in diastolic blood pressure after bypass surgery.	(43)

Beyond association studies linking adipocyte hypertrophy with obesity phenotype (tier 1-2), prospective studies demonstrate that adipocyte size predicts subsequent, clinically-relevant, obesity-related outcomes (**Table 1**, tier 4). In Pima Indians who had normal glucose tolerance at baseline, abdominal subcutaneous adipocyte cell size predicted incident T2DM during a mean follow-up of 9.3 years (38). Although adipocyte cell size correlated with insulin resistance, the two factors contributed independently to incident T2DM risk: The risk of developing T2DM among those with abdominal subcutaneous adipocytes in the 90^th^ size percentile was 5.8-fold greater than those in the lower 10 percent, after adjusting in the multivariate Cox regression analysis for sex, age and percent body fat (which were non-predictive of T2DM), and for insulin sensitivity and acute insulin response to a glucose load, which were also identified as independent predictors of incident T2DM (38). This finding was then corroborated by a study that followed 234 Swedish women over a 25 year period (39). A stepwise multivariate model adjusted for age, family history of T2DM and waist-to-height ratio (the strongest independent predictor in this study), demonstrated a hazard ratio of 1.54 for every standard deviation increase in abdominal subcutaneous adipocyte size. Interestingly, femoral subcutaneous adipocyte size did not remain an independent predictor of T2DM in the multivariate models, despite correlating with abdominal subcutaneous adipocyte size (39).

Prediction of obesity intervention outcome by adipocyte size assessment was proposed by a 2-center, French-German study (20). Though statistical models fell short of detecting a strong independent prediction, an association was found between subcutaneous mean adipocyte size and resolution of metabolic risk/dysfunction: Six months after bariatric surgery, women whose composite phenotype of T2DM or a high risk for developing T2DM had been resolved had smaller mean adipocyte size at baseline, compared to women whose metabolic dysfunction did not resolve postoperatively (20). A different result was obtained in a Swedish study that observed greater improvements in insulin sensitivity following weight loss among those with hypertrophic subcutaneous adipocytes at baseline (43). Two cohorts were examined to explore this relationship in response to moderate (~7%) weight loss (induced by dietary intervention, n=100), or more pronounced weight loss (33%, induced by gastric bypass surgery, n=61). Baseline adipocyte size per-se positively correlated with the subsequent improvement in insulin sensitivity (delta-HOMA-IR) only in the surgery intervention group. Yet, when adipocyte morphology value was considered – an index of adipocyte volume to total fat mass used to dichotomously classify patients to those with hyperplastic versus hypertrophic adipocytes (further discussed below) – this association was evident in both cohorts. In addition to greater improvement in insulin sensitivity, larger adipocytes predicted greater reductions in diastolic blood pressure, but not in blood lipid parameters (43). Importantly, other baseline clinical parameters, such as anthropometric measurements, did not predict the degree of metabolic improvement in response to weight loss intervention, suggesting the potential unique clinical value in assessing this OrAD feature.

Jointly, current studies assessing the possibility to predict obesity-related outcomes by adipocyte size are promising, but seem too few to propose clear guidelines before additional studies are available. Notably, tier 4 studies (**Table 1**) suggest that larger subcutaneous adipocytes may predict different outcomes in different clinical settings: In observational prospective studies they may reflect higher risk of future metabolic deterioration; In acute, experimental weight-gain settings, larger subcutaneous adipocytes may predict less weight gain and a lower decline in insulin sensitivity; As predictors of post-bariatric/metabolic surgery, results are inconsistent, and may also require single-center assessment of adipocyte size. Finally, although subcutaneous and visceral adipocyte size are correlated, they may have differing predictivity of clinical endpoints. Since visceral fat biopsies are only available in patients undergoing abdominal surgery, current information is more limited regarding the association between visceral adipocyte size and subsequent clinical endpoints.

#### Additional Considerations Related to Adipocyte Size Assessment for Clinical Applications

- *Which fat depot?* Visceral (mainly studied is omental) adipose tissue is considered to be already a pathogenic/ectopic depot (2), and is therefore more intuitively connected to OrAD. Indeed, omental adipocyte size was repeatedly associated with metabolic (14, 15, 18, 19, 46, 47, 52) and cardiovascular dysfunction (42, 46). However, as described throughout this section, subcutaneous adipocyte size also correlates with these outcome measures – albeit to a lesser degree (15, 47, 52). Additionally, subcutaneous adipose tissue is clinically-accessible by a percutaneous biopsy, a minimally-invasive procedure under local anesthesia. Such procedure is reminiscent not only of percutaneous biopsy of solid tumors, but also of liver biopsy that is quite routinely preformed in the hepatology clinic. Six of the seven tier 4, predictive studies (**Table 1**) demonstrate that abdominal subcutaneous adipocyte size may also be predictive of obesity-related outcomes. Possibly, this relates to the fact that subcutaneous adipose tissue constitutes the largest fat depot in the human body, so merely by its mass, changes in this depot might exert significant impact at the whole-body level. However, even within subcutaneous adipose tissue anatomical location may impact the effect of increased adipocyte size, as abdominal subcutaneous adipocyte size was found superior to femoral adipocyte size estimation in predicting incident T2DM (39). The possible predictive value of adipocyte size in other depots such as omental adipose tissue, and even in sub-compartments of subcutaneous fat [superficial and deep subcutaneous fat, whose mass is differently associated with obesity-related morbidity (53)], require further research.

- *Method for determining adipocyte size:* Adipocyte size measured (estimated) by histological sections is likely the most available approach in most clinical settings, as it can be analyzed manually or in a semi-automated manner using image analysis software by a pathologist (**Box 1**). Yet, this clearly provides an (under)estimation of true adipocyte size, rather than an absolute size determination, and has additional biases detailed elsewhere (10, 44). Other methods include microscopic assessment of isolated adipocytes obtained by collagenase digestion of fresh tissue, osmium tetroxide-fixed isolated adipocyte size estimation, and scanning electron microscopy. Each of these approaches has its potential biases (10, 44, 55), and may be limited to centers with specialized labs that have established the technique. In general, the different methods do correlate quite well with each other, and, cross-sectionally, with adiposity measures (52). Yet, absolute size determination is crucial if one also seeks to calculate adipocyte number (55), which may be an independent parameter that associates with obesity-related endpoints.

*Adipocyte size versus adipocyte morphology, and adjustment approaches -* Although adipocyte size – diameter or calculated volume – has been used and shown to associate with obesity-related phenotypes, adipocyte morphology value may be a stronger predictor (43). This parameter is derived from the curvilinear association between adipocyte volume and total body fat mass (19, 44, 56), and subtracting the expected adipocyte volume from the actual measured adipocyte volume. An adipocyte morphology value above the expected denotes hypertrophy, whereas a negative value (i.e., below expected) denotes hyperplasia. Studies have also reached different conclusions on whether adipocyte size associates with clinical parameters independently of (i.e., when adjusted for) adiposity measures. This may be attributed to the adjustment for different adiposity measures (BMI, total body fat mass, regional fat, etc.). Since several studies showed that abdominal subcutaneous adiposcyte size was no longer associated with insulin resistance following adjustment for visceral fat volume, Tchernof et al. proposed that “excess visceral adipose tissue accumulation and subcutaneous fat cell hypertrophy may represent markers of a common phenomenon: limited hyperplasic capacity of adipose tissues*“* (44).

*Which size parameter should be looked at?* Adipocyte size may not be normally distributed, particularly when adipocyte volume is used (43), and bi-modal and skewed distributions have also been proposed (44, 52). This suggests that perhaps beyond mean (average, or even median) adipocyte size, other adipocyte size measures should be considered, including the maximal adipocyte size, or conversely – the percent of small adipocytes (57), large adipocytes (58), etc. Although measuring the larger and smaller range of adipocyte size may be particularly challenging methodologically (44), such measures may prove to disclose clinically useful associations with obesity and treatment related outcomes.

*What do larger adipocytes mean for adipose tissue biology?* Having larger mean adipocyte size, or an adipocyte morphology above the expected value, defines hypertrophic adipose tissue expansion (43). Yet, it remains controversial how this relates to the adipose expandability theory – i.e., whether it reflects decreased or rather improved capacity of adipose tissue to expand in response to excess calories, and protect from ectopic fat accumulation and insulin resistance. In fact, some studies suggest that higher mean adipocyte size but lower fraction of large adipocytes (higher percentage of small adipocytes), may actually represent impaired capacity to retain metabolic health in response to overfeeding or obesity (40, 41, 58). This was attributed to decreased lipogenic/adipogenic capacity (of the small adipocytes), and to a pro-inflammatory skeletal muscle response, possibly secondary to rapidly hypertrophying small adipocytes.

### Adipose Tissue Inflammation

The link between obesity and adipose tissue inflammation had been extensively studied over the past 25 years, initially implicating obesity-associated changes in adipose tissue cytokines and later adipose tissue immune cells [references (59) and (60, 61) are considered as milestone studies that sparked research in these directions, respectively]. Excellently reviewed in recent years (62, 63), adipose tissue inflammation engages multiple arms of the immune system, with marked alterations in inflammatory gene expression and immune cell populations. Initially (and still largely) considered to offer a (causal) link between obesity and the development of adipose tissue and whole-body metabolic dysfunction and increased cardiometabolic risk, in reality this link is largely more complex, and less established in humans than in rodent models. Not only causality may be bi-directional (64), inflammation in general, and specifically in adipose tissue, cannot be simplistically viewed only as a pathological process, but as a dynamic process that is ignited in response to multiple perturbations, with the initial aim of restoring homeostasis. In established obesity, the chronic nature of low-grade adipose tissue inflammation, which in humans sometimes lasts decades, may be a major feature that is not necessarily fully captured by rodent studies. Indeed, pre-clinical and some clinical studies questioned the putative causal association between adipose tissue inflammation and metabolic dysfunction in obesity. Complementarily, adipose tissue inflammation may be in some instances a beneficial phenomenon of adipose tissue remodeling that eventually contributes to improved whole-body metabolic health and homeostasis, particularly in the early response to excessive weight loss.

Cross-sectionally, multiple human studies demonstrated activation of various inflammatory pathways in adipose tissue of patients with obesity versus people without obesity. Beyond lean-obese comparison (i.e., within the obese population), cross-sectional studies provide links between a higher pro-inflammatory state, particularly in visceral adipose tissue, and greater obesity-related cardiometabolic complications (selected studies are presented in **Table 2**, tier 1). Yet, even at this tier, some immune cells (e.g. macrophages) have been shown to positively associate with metabolic dysfunction, while others (e.g. mast cells) exhibited negative association (i.e., higher abundance of mast cells associated with better metabolic profile). Longitudinal co-association studies [Comprehensively reviewed in (82), and specifically in the response to bariatric surgery in (84), and selected publications presented in **Table 2**, tier 2], have somewhat surprisingly shown even more inconsistencies: Improvements in metabolic dysfunction following lifestyle, pharmacological or surgical interventions were associated with relatively mild, or even without any decline in adipose tissue inflammation parameters. Here, again, which inflammatory parameter was assessed, what was the intervention, and particularly follow-up duration, are likely major determinants of the results and conclusions. Overall, tier 2 studies may suggest that early improvement in metabolic dysfunction following weight loss may not require resolution of adipose tissue inflammation. In the excessive weight-loss response to bariatric surgery, some inflammatory markers (neutrophil or macrophage infiltration and related chemokines and cytokines) may even be increased up to 6 months postoperatively, followed by a gradual decline (85). An additional ‘uncoupling’ between adipose tissue inflammation and insulin resistance was demonstrated in the response to experimental weight gain, which induced insulin resistance without apparent stimulation of systemic or adipose tissue inflammation (81).

**Table 2:** OrAD feature - Adipose tissue inflammation.

		References
**Tier 1**: Cross-sectional association *Adipose tissue inflammation correlates with obesity-related diseases*	*Adipose tissue macrophages:*	
	- Higher macrophage abundance in visceral adipose tissue correlates with NAFLD	(65, 66)
	- Higher macrophage abundance in visceral adipose tissue correlates with insulin resistance or metabolic dysfunction.	(15, 65, 67, 68)
	- Larger number of macrophage “crown-like structures” in subcutaneous fat in women with obesity and T2DM Vs. NGT.	(69)
	*Adipose tissue mast cells:*	
	- Higher visceral adipose tissue mast cell abundance associates with better (among patients with obesity)//worse (when also compared to non-obese patients) metabolic profile.	(70, 71)// (72)
	*Adipose tissue dendritic cells (DC):*	
	- Higher abundance of CD11c+/CD1c+ DCs in subcutaneous fat correlates with insulin resistance in morbidly obese patients.	(73)
	*Adipose tissue T-cells:*	
	- Higher abundance of CD4+ (Th1, Th17) and CD8+ T-cells in both subcutaneous and visceral adipose tissues in obesity associate with insulin resistance.	(74)
	*Expression of inflammatory genes:*	
	- Higher expression of inflammatory genes and/or lower expression of anti-inflammatory genes associate with insulin resistance and/or high cardiometabolic risk in obesity.	(68, 74, 75)
**Tier 2**: Longitudinal co-association *Adipose tissue inflammation changes during intervention associate with changes in obesity-related phenotype/risk*	*Weight loss* The magnitude of weight loss following bariatric surgery correlates with *decreased* adipose tissue:	
	- macrophages;	(70, 76, 77)
	- mast cells;	(70)
	- expression of inflammatory cytokines	(70, 77)
	Weight loss following bariatric surgery or lifestyle intervention, and consequentially improved metabolic profile, *do not associate with decreased* (and even increased):	
	- subcutaneous adipose tissue macrophages and other leucocytes	(78–80)
	- inflammatory gene expression	(78–80)
	*Experimental weight gain*	
	- Experimental acute weight gain induces insulin resistance without activating systemic or adipose tissue inflammation.	(81)
**Tier 3**: Main proposed mechanisms *(human studies)*	- Contribution to systemic inflammation.	(82)
	- Decrease in cardiovascular-protective, adipose derived factors (e.g. adiponectin).	(83)
	- Adipose tissue and whole-body insulin resistance.	(62, 82)
	- Source of inflammatory lipid mediators.	(82)
**Tier 4**: Prospective/predictive studies *Baseline adipose tissue inflammation parameters predicts subsequent intervention outcome*	Higher adipose tissue expression of mast cells -specific genes predicts greater weight-loss response to bariatric surgeries *(cohort 1: n=18, 6 months follow-up, only visceral adipose tissue; cohort 2: n=56, 1y follow-up, both visceral and subcutaneous adipose tissue)*	(70)

Nevertheless, several mechanisms have been proposed to explain the apparent link between adipose tissue inflammation and obesity-related complications, most notable of which is adipose tissues’ contribution to systemic inflammation and obesity-related decline in vascular-protective adipokines (e.g. adiponectin, **Table 2**, tier 3). Yet, such mechanistic propositions are frequently indirect. Clinically, attempts to relieve obesity-related cardiometabolic complications (i.e., T2DM and related elevated cardiovascular risk) using available anti-inflammatory interventions has raised high hopes (86, 87) but was largely met with somewhat limited results. These call, again, for the need to better understand adipose tissue inflammation and its specific mediators that may link to obesity-related complications, and/or to find biomarkers that can identify humans with a defined obesity sub-phenotype, who would benefit from specific anti-inflammatory intervention(s) – i.e., predictive (tier 4) studies.

In light of this apparent need, it is surprising that while a relatively rich body of literature examines cross-sectionally or longitudinally the association between parameters of adipose tissue inflammation and obesity phenotypes, tier 4 predictive studies are surprisingly scarce (**Table 2**, tier 4). In two independent cohorts we have shown that higher expression of mast cell specific genes, which we found to be indicative of adipose tissue mast cell abundance assessed histologically, predicted the degree of subsequent weight loss induced by bariatric surgery (70). In the Israeli cohort (n=18), those who expressed mast cell -specific chymase CMA-1 above the median exhibited weight loss that was nearly 2-fold greater than those who were “CMA-1-low”. CMA-1 expression in subcutaneous adipose tissue also predicted greater weight loss 1-year post surgery in an independent, German cohort (n=56). In this cohort, in omental adipose tissue, other mast cell genes [tryptase 1 (TPSB1) and c-kit (KIT)] positively correlated with the degree of weight loss. Despite extensive literature search, we could not find additional tier 4 predictive studies that explore whether baseline markers of adipose tissue inflammation correlate with future obesity-related endpoints and intervention outcomes. This surprising paucity in the literature is further discussed in the final section of this review.

### Adipose Tissue Fibrosis

Fibrosis is the pathological deposition of extracellular matrix (ECM) in a tissue, such that it replaces portions of the tissue parenchyma, changes the tissue’s physical properties, and impairs its function.

This elaborate process is reviewed in detail in references (54, 88, 89). Briefly, while the ECM in healthy adipose tissue is constantly being deposited and degraded as part of normal adipose tissue homeostasis, when excessively deposited, adipose tissue becomes fibrotic. As discussed in the previous sections, obesity manifests with pathological adipose tissue expansion. Adipocyte hypertrophy is associated with adipocyte cell death and inflammation, which trigger dysregulated ECM deposition and fibrosis. Moreover, increased cross-linking of ECM proteins in obesity stiffens adipose tissue and exerts mechanical pressure on hypertrophied adipocytes, resulting in further adipocyte death, lipid spillover and exacerbation of tissue and systemic inflammation.

Cross sectional association studies in humans with obesity (**Table 3**, tier 1) show that increased degree of obesity and fat mass associate with increased adipose tissue fibrosis (90) and non-alcoholic fatty liver disease (NAFLD) (96). However, conflicting evidence exist regarding how adipose tissue fibrosis associates with metabolic dysfunction in obesity. Although some studies suggest that decreased fibrosis in both subcutaneous and visceral (omental) fat depots is associated with at least some aspects of metabolic disease (98, 99), most reports provide evidence that metabolic dysfunction (e.g., insulin resistance) associates with increased adipose fibrosis, both in subcutaneous (91, 94, 106, 107) and omental (94, 95) adipose tissues.

**Table 3:** OrAD feature - Adipose tissue fibrosis.

		References
**Tier 1**: Cross-sectional association *Adipoce tissue fibrosis correlates with obesity-related disease endpoints and/or cardiometabolic risk*	*Associated with increased adipose tissue fibrosis:*	
	- Increased adiposity (obesity fat mass).	(90–93)
	- Insulin resistance.	(91–95)
	- NAFLD/Increased liver fibrosis	(96)/ (97)
	*Associated with decreased adipose tissue fibrosis:*	
	- Insulin resistance/Type 2 diabetes	(98)/ (99)
**Tier 2**: Longitudinal change association *Adipose tissue fibrosis changes during intervention -/+ association with changes in obesity-related phenotype/risk*	*Decreased adipose tissue fibrosis:*	
	- With weight loss (even as low as 5%)	(80, 90, 92, 100)
	*No change in adipose tissue fibrosis:*	
	- Following weight loss (bariatric surgery) despite metabolic improvement	(94, 101)
	*Experimental weight-gain:*	
	- Increased adipose tissue fibrosis following 8 weeks of overfeeding-induced weight gain	(90)
**Tier 3**: Possible mechanisms *(human studies)*	- Increased inflammation, reduced lipid storage capacity and lipid spillover to ectopic fat deposits	Reviewed in (88, 102, 103)
	- Col6A3-related decreased adipose tissue oxygenation	(104)
**Tier 4**: Prospective/predictive studies *Baseline adipose tissue fibrosis predicts intervention outcome*	- Higher baseline total and pericellular fibrosis in subcutaneous adipose tissue associated with less fat mass loss after 3, 6 and 12 months post-bariatric surgery *(n=65 persons with obesity)*.	(98)
	- Greater total subcutaneous adipose fibrosis adjusted for age, diabetes and circulating IL-6 was associated with a low weight loss response (i.e., <25% decrease in BMI). *(n=243 persons with obesity*, *OR [95% CI] = 1.58 [1.10-2.28]).*	(97)
	- Histological adipose fibrosis score (FAT) predicts weight loss following RYGB bariatric surgery: Pre-operative FAT score ≥2 was associated with 3-fold increased risk of reduced weight loss 12 months following the surgery *(n=183 persons with obesity)*.	(105)

Longitudinal studies in humans (**Table 3**, tier 2) show that fibrotic markers increase post experimental weight gain (90), and decrease with weight loss (80, 92, 100). Interestingly, even moderate 5% weight loss induced by lifestyle modification was sufficient to reduce the expression of several ECM genes in adipose tissue, whereas inflammatory gene expression did not change (80). This suggests that during moderate weight fluctuations, metabolic changes may be more closely associated with changes in fibrotic gene expression in adipose tissue than with markers of adipose tissue inflammation. Nevertheless, in response to more pronounced weight loss, such as following bariatric surgery, adipose tissue fibrosis was not reduced despite improvements in metabolic outcome measures (i.e., decrease insulin resistance) (94, 101, 108). Possibly, this is reminiscent of the effect reported by some studies in response to bariatric surgery on adipose tissue inflammation in the first 6 month (**Table 2**, tier 2).

Proposed mechanisms (**Table 3**, tier 3) implicate hypoxia and/or activation of hypoxia inducible factor 1 (HIF-1) as central mediators between adipose tissue fibrosis and metabolic complictions of obesity [reviewed in (88, 102, 103)]. Of particular interest is adipose tissue Collagen 6A3 (Col6A3), which correlates with adiposity (BMI and fat mass) (90, 91), metabolic dysfunction (91), and with reduced PPARϒ expression. Evidence suggests that Col6A3 acts to propagate a fibro-inflammatory phenotype by promoting reduction in tissue oxygenation and inducing HIF-1α expression (104), while PPARγ activation inhibits Col6A3 expression (69, 73) and promotes adipogenic differentiation, adipose tissue vascularization and suppression of HIF-1α (73).

Beyond association studies, several human studies demonstrate that assessing the degree of adipose tissue fibrosis predicts subsequent clinically-relevant outcomes of intervention in persons with obesity (**Table 3**, tier 4). In a comprehensive effort to accurately characterize fibrosis in different adipose depots and define its clinical relevance, 9 healthy-weight controls and 65 patients with obesity, who met the criteria for bariatric surgery (BMI >40 or >35 kg/m^2^ with at least one comorbidity), were recruited. Participants were analyzed before, and 3, 6 and 12 months postoperatively. Preoperative total and pericellular fibrosis in subcutaneous adipose tissue, measured by picrosirius red staining, correlated with percent fat loss at 3, 6 and 12 months postoperatively (Total fibrosis: 3 months, R=-0.39, p<0.005; 6 months, R=-0.31, p<0.05; and 12 months, R=-0.30, p<0.05. Pericellular fibrosis: 3 months, R=-0.23, p<0.05; 6 months, R=-0.32, p<0.05; and 12 months, R=-0.30, p<0.05) (98). After clustering the participants into 3 groups based on the percent fat loss (using a k-means algorithm), baseline pericellular fibrosis was significantly higher in cluster C, in which participants lost the least weight postoperatively. A later study by the same group, examined a sub-cohort of 243 out of 404 bariatric surgery patients at baseline, and 3, 6, and 12 months postoperatively (97). Biopsies were collected during surgery, and used to assess fibrosis in liver, subcutaneous and omental adipose tissues by picrosirius red staining. Adipose tissue fibrosis in both subcutaneous or omental depots were associated with liver fibrosis and measures of adiposity (body weight, percent fat and BMI). Participants were further divided into ‘good responders’ (GR), who lost >34.8% of baseline BMI 12 months after surgery, and ‘less responsive’ (LR), who lost <25%. In a multivariate analysis, higher total subcutaneous adipose tissue fibrosis adjusted for age, diabetes and circulating IL-6 characterized the LR group (OR [95% CI] = 1.58 [1.10-2.28]) (97). These data suggest that subcutaneous adipose fibrosis can be used to predict weight loss outcome of bariatric surgery. This can assist in improving the preoperative expectation from the surgery, and if bariatric surgery is performed, to consider intensified postoperative intervention to optimize the weight-loss response to the surgery (as further discussed in subsequent section of this review). Indeed, for such clinical purposes, histology-based tool to grade the degree of adipose tissue fibrosis from surgical biopsies was established, and exhibited successful prediction of weight loss following bariatric surgery in 183 patients with severe obesity (105): Fibrosis score of Adipose Tissue (FAT score) is a semiquantitative tool that uses subcutaneous adipose surgical biopsies to evaluate adipose tissue fibrosis following picrosirius red staining. It utilizes a 4-stage fibrosis rating scale where Stage 0=no apparent perilobular (PLF) or pericellular (PCF) fibrosis; Stage 1= moderate PLF and/or PCF; Stage 2= severe PLF or severe PCF; and Stage 3 = severe PLF and severe PCF. FAT score was associated with adipose tissue fibrosis and with increased M2 macrophage infiltration, but not with adipocyte size. Importantly, higher FAT score was correlated with lower weight loss 12 months following bariatric surgery, and a baseline FAT score ≥2 was associated with a 3-fold increased risk of reduced weight loss response to bariatric surgery (OR [95% CI] = 3.2 [1.7 – 6.1]). When testing the ability of the FAT score to predict weight loss post bariatric surgery, the authors compared 3 prediction models and concluded that there is merit and added value in incorporating assessment of subcutaneous adipose pathology in prediction of weight loss response to bariatric surgery (105).

It is noteworthy that all 3 tier 4 studies (97, 98, 105) were by K. Clément and co-workers, representing the development and experience of a single center. Clearly, given the possible impact of the findings, it is imperative that these results are replicated and reported by additional centers, so that analysis of adipose tissue fibrosis could be considered as part of the routine clinical toolkit in the treatment of obesity.

#### Additional Considerations Related to Adipose Fibrosis Assessment for Clinical Applications

- *Which fat depot(s)?* The few studies that assessed fibrosis in both subcutaneous and omental adipose tissue reached conflicting conclusions: While Muir et al., report no differences in adipose fibrosis assessed using both picrosirius red staining and fibrotic gene expression between subcutaneous and omental depots (99), Divoux and colleagues demonstrate different collagen patterns between the 2 tissues (98). Moreover, these authors report increased total fibrosis percent (picrosirius red staining) in participants with obesity compared to non-obese controls in subcutaneous, but not omental fat.

- *Method for obtaining adipose tissue biopsy:* The two most common techniques to obtain adipose tissue samples are surgical biopsies and needle-aspirated biopsies – each has its own pros and cons. A recent study compared the two techniques side by side and found that the biopsy technique affects adipose tissue gene expression profile. Specifically, needle-aspirated core biopsies were more fragmented due to the mechanical shear stress applied while the tissue is forced through the needle. As a result, needle-aspirated biopsies do not effectively aspirate the fibrotic fraction of subcutaneous adipose tissue, contain an unrepresentative smaller stroma-vascular fraction and thus misrepresent adipose tissue fibrosis (109).

- *Emerging imaging approaches to assess adipose tissue fibrosis:* A few non-invasive approaches have been proposed for assessing adipose tissue fibrosis. Transient Elastography provides assessment of adipose tissue stiffness by measuring the shear wave velocity of the tissue’s response to vibration, correlating with the degree of fibrosis assessed histologically (98, 110). Recently, a new MRI application was validated against adipose tissue histology, successfully quantifying fibrosis in subcutaneous adipose tissue (111). Potentially, by creating a 3D fibrosis assessment of the subcutaneous adipose tissue, this approach may be less prone to sampling biases, which is common in histological assessment, particularly of biopsies obtained by needle aspiration.

*- How fibrosis is quantified:* Percent pericellular and percent peri-lobular fibrosis might be more sensitive measurements compared to total percent fibrosis, and may therefore prove to provide more clinically relevant information. For example, when comparing fibrosis in subcutaneous and omental adipose tissues from participants with obesity and non-obese controls, percent pericellular fibrosis, but not percent total fibrosis, differed between the groups in both depots (98).

- *Patients’ characteristics might impact adipose tissue fibrosis:* Differences in adipose tissue fibrotic response to obesity and weight loss exist between different ethnic groups, for example Caucasian vs Asian-Americans (112) or Asian-Indian (113). Some studies show sex differences in adipose fibrosis (90), and aged individuals exhibit increased adipose tissue fibrosis [reviewed in (114)].

## Incorporating OrAD Assessment in the Obesity Clinic

There is a growing “need to go beyond BMI” notion in clinical obesity medicine in the quest for better personalizing the treatment of people with obesity, and assessment of the health of the adipose tissue is certainly a potential avenue. Hence, in previous sections we provided examples for clinically-valuable information obtainable from mostly standard pathology assessment of adipose tissue biopsies in order to use such information as a decision-making tool. We hereby describe how adipose tissue biopsies and OrAD assessment could be incorporated into the obesity clinic, with relevance to patients undergoing bariatric/metabolic surgery, those offered non-surgical interventions, and even as part of the decision-making process between these two options. We consider both primary and secondary prevention concepts related to obesity and its related complications.

*Adipose tissue biopsies during bariatric/metabolic surgery –* When a person with obesity undergoes bariatric surgery (or for that matter, any elective intra-abdominal surgical procedure), sampling abdominal subcutaneous and omental adipose tissue could easily be incorporated as a standard care procedure. This is because, first and foremost, adding an excision of a few grams of adipose tissue is surgically simple, fast, and runs nil or very low added risk for the patient. Conceptually (medically), it is equivalent to sampling the diseased tissue in other medical circumstances, such as in oncological surgeries. As discussed above, rather standard histopathological assessment (**Box 1**) can already provide enormous amount of information, and does not require a special setting beyond that available in most operating rooms – placing the biopsies immediately in formaldehyde, and sending to the affiliated department of pathology.

**Box 1 Common/standard clinical laboratory methods available to assess OrAD.**

**Table:** 

Feature	Method/approach	Comments	Clinical lab	Reviewed in ref
**A.** Adipocyte size	Histological estimation of adipocyte size: H&E staining *- manual counting of adipocytes/field.* *- automated (software) assessment of histological images.* Isolated adipocytes - *manual microscopic assessment of isolated cells.* *- Automated cell size analyzer.*	requires collagenase digestion of adipose tissue	Pathology Pathology/cytology	(10, 44)
**B.** Adipose tissue inflammation	Histopathology: *- H&E staining – assess leucocyte infiltration based on cellular overall morphology* *Immunohistochemistry using immune cell -specific antibodies:* * - anti-CD45 (common leucocyte antigen)* * - anti-CD68 staining (macrophages)* * - anti-CD117 (c-kit), tryptase (mast cells)* * - anti-CD3 (T-lymphocytes)* * - anti-CD20 (B-lymphocytes)* * - anti-CD57 (NK cells)* *Flow cytometry of stromal-vascular cells (Note: adipocytes are usually unamenable for flow cytometry analysis)*		Pathology Cytology; hematology	
**C.** Adipose tissue fibrosis	Histopathology: *- H&E staining* *- Masson Trichrome staining* *- Picrosirius red -> “FAT score”* *- Collagen immunostaining (immunohistochemistry using anti-collagen antibodies)*	May be non-standard assay	Pathology	(54)

Adipocyte cell size, amount of fibrosis and degree of inflammation can all be evaluated on a paraffin block section stained with standard hematoxylin & eosin (H&E). The main advantage of this method is its availability practically in all medical centers (which have a pathology department). Additionally, this technique allows examination of tissue architecture and performance of histochemical and immunohistochemical stains. A. Adipocyte size can be measured either manually or by using automated cell size analyzer on cytology specimens. Additionally, adipocyte cell size can be estimated manually on H&E-stained slides according to the average amount of adipocytes per 10 high-power fields (HPFs); one can also use software analysis for this purpose. B. Degree of Inflammation can be evaluated based on H&E-stained slides. Immunohistochemical stain for CD45 (Leukocyte Common Antigen) would highlight all inflammatory cells, making this estimation easier and possibly more accurate. In addition, identification of specific inflammatory cells can be achieved using immunohistochemical stains: CD68 for macrophages, CD3 for T-lymphocytes, CD20 for B-lymphocytes, CD57 for NK cells and CD117 (c-Kit) or tryptase for mast cells. **C.** Amount of fibrosis can be assessed based on H&E-stained sections or preferably using Masson’s Trichrome stain that would highlight connective tissue fibers, staining them in a blue hue. Picrosirius red and immunohistochemical stain for specific collagens would also emphasize collagen fibers.

*Subcutaneous adipose tissue needle biopsy or minimally invasive, small open surgical biopsy in the non-surgical patient with obesity* – This clinical setting is more complex, as it requires subjecting patients to a procedure which is still non-standard in most clinics. Subcutaneous (usually abdominal, at the lower quadrant) adipose tissue biopsy is minimally-invasive, is performed under local anesthesia, but even if of very low risk for the patient does run added risk of local hematoma, infection, and pain. Conceptually, subcutaneous adipose tissue needle biopsy can be viewed similarly to liver biopsy in the clinical workup of patients with liver disease, and is associated with lower risks. Yet, while as discussed above in this review, such procedure can impact therapeutic options by improving the prediction of expected outcomes, it is still investigational, and requires institutional ethical committee approval, patients’ written informed consent, and careful personalized cost-benefit assessment.

Below are a few example scenarios for how might OrAD assessment inform the obesity clinician and improve precision care.

*Predicting post-operative weight loss response*- After bariatric/metabolic surgery, OrAD features indicative of predicted low postoperative weight-loss response to the bariatric surgery [high SC fat fibrosis score (105), low omental fat mast cell count (70), possibly adipocyte cell size (20)], may suggest the consideration of intensified post-operative intervention to ensure optimal weight loss. Possibilities may include intensifying post-operative life-style interventions and/or pharmacotherapy. Once more data are available – both on OrAD and on long-term follow-up endpoints, it would be important to determine if OrAD features can predict failure to maintain long-term weight loss induced by bariatric surgery (**Box 2**), as it is increasingly recognized that over a third of patients regain >25% of their maximal postoperative weight loss (115).

**Box 2: Major outstanding questions**

- Can OrAD features predict intervention outcomes:
- response to emerging anti-obesity pharmacotherapy;
- a-priori identify successful weight-loss responders to incretin receptor agonists;
- post-bariatric/metabolic surgery weight regain.
- “Composite OrAD score” – would a composite score that integrates different OrAD features improve predictability of obesity-related endpoints and/or intervention outcomes?
- Reversibility of OrAD features in the response to intervention: Could a second, early post-intervention initiation OrAD assessment serve to predict long-term successful response?
- Could repeated OrAD assessment of the metabolically-healthy patient with obesity identify pending metabolic/health deterioration?
- Could OrAD assessment predict less-conventional obesity-related risks, such as obesity-related cancer, cognitive decline, etc?
- Is OrAD a marker or a therapeutic target on its own? Would improving OrAD features improve obesity related outcomes?

*Predicting postoperative effect on cardiometabolic risk* - OrAD features indicative of lower chances of reducing obesity-related cardiometabolic risk following bariatric surgery could support considering interventions aimed at secondary prevention. Although suggested to predict incident T2DM in observational studies (38, 39), the predictive value of adipocyte size assessment in the post-bariatric surgery patient is still unclear (**Table 1**, tier 4). Moreover, when attempting to predict postoperative diabetes remission, adipocyte size was not found to differ between patients with obesity and T2DM who exhibited 1y postoperative diabetes remission or not (116). Yet, a current debate is whether to discontinue all anti-diabetic medications including metformin after bariatric/metabolic surgery in the post-operatively normoglycemic patient. Possibly, a higher OrAD burden may provide a rationale for postoperative continuation of metformin.

*Primary prevention in patients with “metabolically-healthy” obesity –* Subcutaneous adipocyte size had been shown to predict deterioration of glycemic control even in patients with normal glucose tolerance, and beyond other risk factors (38, 39). Although further demonstration in other cohorts would be valuable, existing studies already suggest such association in diverse populations. Hence, larger adipocyte size [which may require threshold assessment in the specific clinic (20)] may indicate a need for intensified measures for primary prevention of metabolic deterioration. Collectively, in this setting, assessing OrAD in subcutaneous adipose tissue can assist in refining/sub-classifying the metabolically-healthy obese phenotype, highlighting those at greater cardiometabolic risk despite their current intact metabolic profile.

Additional outstanding questions that could inspire future studies to assess the clinical value of OrAD assessment are presented in the following section and in **Box 2**.

## Outstanding Questions, and Future Directions

In modern medicine, sampling the diseased tissue in order to extract clinically-useful information that directs treatment is a common practice. We hope that this review conveys the message that adipose tissue in obesity is a potentially rich source of clinically-meaningful information that should be utilized in the hope of improving precision medicine for the care of patients with obesity. Studies that actually demonstrate that routine assessment of OrAD features could guide personalized care by providing prediction of prognosis and/or response to anti-obesity intervention are still too few to be translated into clinical guidelines. But they do provide a proof of principle that is sufficient to motivate joint efforts to extract such clinically-useful information. Standard laboratory techniques available in most centers, particularly pathology labs, can already provide enormous amount of information on OrAD features, which then needs to be linked to relevant endpoints and post-intervention outcome assessment (**Box 1**). There is no doubt that predicting intervention outcome(s) will become an increasing need with newly-emerging options for anti-obesity treatments in the upcoming future.

### Molecular Signatures of Adipose Tissue

This review focused on histopathological assessment of OrAD, but molecular tools should also be considered. Indeed, multiple molecular differences between people without or with obesity have been reported, and have greatly contributed to current understanding of OrAD. Yet, fewer studies assessed, even cross-sectionally, molecular differences within the heterogenous group of persons with obesity in the quest to explain obesity sub-phenotypes (15, 75). Molecular OrAD patterns associated with longitudinal changes were shown using both coding and non-coding (micro-RNA) RNAs. For example, successful maintainers of weight loss exhibited an increase in oxidative phosphorylation gene expression, and a decrease in cell-cycle and inflammatory genes in subcutaneous fat, while unsuccessful weight loss maintainers exhibited the opposite gene expression change (117). Using micro-RNAs, changes in subcutaneous adipose tissue expression of several miRNAs associated with the magnitude of decreased weight, waist circumference or fat mass loss, with no difference between different dietary intervention strategies (118). Moreover, prospectively, the viscera/subcutaneous fat expression ratio of miRNA-122 predicts the degree of weight loss 1y after bariatric surgery (n=61) (5).

Modern ‘omics’ technologies will clearly advance the information one would be able to extract from any biological sample, including adipose tissue biopsies. Various whole tissue ‘omics’ approaches hold the promise of discovering novel outcome predictors in an unbiased manner. One pioneer attempt tested the ability to *a-priori* predict the response to a 10w hypocaloric diet by a single, snap-shot assessment of global subcutaneous adipose tissue gene expression using microarrays (6). Global gene expression was able to differentiate between the groups with a maximal prediction accuracy (depending on the model used) of 61%. A stringent identification of differential gene expression between subsequent responders (who lost 8-12 kg) and non-responders (who lost up to 4 kg) using 3 statistical approaches uncovered 9 validated differentially-expressed genes among the n=53 microarrays. Yet, using these genes to predict response did not improve the maximal accuracy (of ~80%), that was obtained by using the 34 differentially-expressed genes identified when relieving the FDR criterion to 8% in the Significance Analysis of Microarray (SAM) procedure. The authors of this study concluded that the predictive performance of this approach was insufficient to be helpful in the clinical setting, and suggest that perhaps a second sample aimed at assessing the initial response to the intervention could better predict the final response to the intervention (6).

Finally, single-cell transcriptomics technologies, such as single-cell RNA-sequencing of the stromal-vascular cells of adipose tissue, or single-nucleus RNA-sequencing that can also capture adipocytes, provide new opportunities to uncover the cellular landscape, and cell-type specific gene expression changes in adipose tissue, at an unprecedented resolution (119). These will allow to determine whether specific cellular composition of adipose tissue – either specific cell types, and/or sub-populations of adipocytes - could effectively predict intervention outcome. For example, it’s plausible that greater proportions of functionally-beneficial adipocytes such as adiponectin producing/insulin sensitive, and/or thermogenic adipocytes, may be expected to not only positively affect whole-body metabolic state cross-sectionally, but to also predict lower risk of developing obesity co-morbidities. If this proves to be true, single-nucleus RNA-sequencing could serve to refine the definition of metabolically-healthy obesity, and prevent un-necessary primary prevention interventions for obesity-related comorbidities in such patients.

### “The Liquid Biopsy Concept” – Can OrAD be Estimated by Blood Sampling?

This review proposes histopathological OrAD assessment as a possible tool to better sub-phenotype patients by predicting obesity related clinical outcomes. A plethora of studies attempt to identify circulating biomarkers (metabolites, miRNA and other epigenetic markers) for the same purpose, related or not to OrAD. Yet, assuming that OrAD is a powerful predictor of obesity outcomes, an additional outstanding question is whether it can be estimated by sampling peripheral blood. This approach, reminiscent of the concept of liquid biopsy in oncology, may be particularly important for providing OrAD assessment of the visceral fat depot in the non-surgical patient, and has been previously attempted (120). As one example, peripheral blood monocyte sub-classes were shown to correlate with lipid storage of omental adipose tissue macrophages (121), which in turn were shown to associate (cross-sectionally) with a worse metabolic phenotype in obesity (122). Circulating blood metabolomics could uncover correlates of OrAD features such as adipocyte diameter, and associate with obesity phenotypes (123). Other circulating biomarkers indicative of OrAD and specifically of adipose tissue communication with other organs that determine obesity-related endpoints, such as fatty liver and cardiac health, should undoubtedly be further explored for clinical use. These include, among others, adipokines and circulating extracellular vesicles, which in obesity are likely significantly contributed by adipose tissue. The adipokine adiponectin is one such example: As it is produced solely by adipocytes, particularly by insulin-sensitive cells, circulating adiponectin levels may indicate improved adipose metabolic function (i.e., lower adiponectin reflecting OrAD). Indeed, although the association between circulating adiponectin and cardiovascular morbidity and mortality is complex and may greatly depend on the health status of the investigated population (124), several studies demonstrated that low circulating adiponectin (total or high molecular weight) were independent predictors of incident metabolic syndrome or T2DM (125, 126).

## Conclusion

Both the motivation to increase precision care in obesity medicine, and the logic of attempting to estimate OrAD to predict clinically-relevant outcomes in obesity care, are clear. Current literature provides some proof-of-principle studies for the possibility that OrAD assessment should be better utilized to enhance precision medicine of patients with obesity. Yet, in some cases (particularly adipose tissue inflammation), the scarcity of predictive studies is surprising in light of the relatively large body of association studies. In particular, longitudinal co-association studies seemingly include all the information required for testing a prediction hypothesis. Current literature remains blind to whether such hypotheses were tested and found to be negative, but remained unpublished. Notably, as an exception, it was reported that adipocyte size and morphology at baseline could not distinguish between patients who exhibited post-bariatric surgery diabetes remission (116). Alternatively, association studies that assessed at least two time-points (baseline and follow-up) may have been underpowered to provide significant predictive data. Therefore, to advance this line of research and clinical practice it would be important to: i. conduct prediction analyses on already available datasets; ii. encourage the report of negative findings, particularly of sufficiently statistically-powered studies; iii. consolidating data from different centers to enable analyses on heterogenous and larger patient databases (i.e., perform meta-analyses); iv. Using advanced approaches, such as deep learning and artificial intelligence, to detect predictive algorithms of OrAD-based clinically-important endpoints.

## Author Contributions

YP reviewed the literature, wrote sections of the manuscript, critically reviewed the manuscript. UY reviewed the literature, wrote sections of the manuscript, critically reviewed the manuscript. YH reviewed the literature, wrote sections of the manuscript, prepared figure, critically reviewed the manuscript. NaM reviewed the literature and critically reviewed the manuscript. NiM reviewed the literature, wrote sections of the manuscript, assisted in preparing figure. RS-L prepared **Box 1**, critically reviewed the manuscript. NB reviewed the literature, critically reviewed the manuscript. DD wrote sections of the manuscript, critically reviewed the manuscript. AR prepared the review’s concept, organized writing missions between co-authors, wrote the manuscript, edited all versions. All authors contributed to the article and approved the submitted version.

## Funding

This manuscript was funded by grants from the DFG (German Research Foundation)—Projektnummer 209933838—SFB 1052 (specific projects: B2), and the Israel Science Foundation (ISF928/14 and 2176/19 to AR).

## Conflict of Interest

The authors declare that the research was conducted in the absence of any commercial or financial relationships that could be construed as a potential conflict of interest.

## Publisher’s Note

All claims expressed in this article are solely those of the authors and do not necessarily represent those of their affiliated organizations, or those of the publisher, the editors and the reviewers. Any product that may be evaluated in this article, or claim that may be made by its manufacturer, is not guaranteed or endorsed by the publisher.

## References

[1] van Woerden G van Veldhuisen DJ Manintveld OC van Empel VPM Willems TP de Boer RA. Epicardial Adipose Tissue and Outcome in Heart Failure With Mid-Range and Preserved Ejection Fraction. Circ Heart Fail (2021) 15(3):245–55. doi: 10.1161/CIRCHEARTFAILURE.121.009238 PMC8920003 34935412

[2] Piche ME Tchernof A Despres JP. Obesity Phenotypes, Diabetes, and Cardiovascular Diseases. Circ Res (2020) 126(11):1477–500. doi: 10.1161/CIRCRESAHA.120.316101 32437302

[3] Lotta LA Gulati P Day FR Payne F Ongen H van de Bunt M. Integrative Genomic Analysis Implicates Limited Peripheral Adipose Storage Capacity in the Pathogenesis of Human Insulin Resistance. Nat Genet (2017) 49(1):17–26. doi: 10.1038/ng.3714 27841877 PMC5774584

[4] Zembic A Eckel N Stefan N Baudry J Schulze MB. An Empirically Derived Definition of Metabolically Healthy Obesity Based on Risk of Cardiovascular and Total Mortality. JAMA Netw Open (2021) 4(5):e218505. doi: 10.1001/jamanetworkopen.2021.8505 33961036 PMC8105750

[5] Liao CH Wang CY Liu KH Liu YY Wen MS Yeh TS. Mir-122 Marks the Differences Between Subcutaneous and Visceral Adipose Tissues and Associates With the Outcome of Bariatric Surgery. Obes Res Clin Pract (2018) 12(6):570–7. doi: 10.1016/j.orcp.2018.06.005 29960868

[6] Mutch DM Temanni MR Henegar C Combes F Pelloux V Holst C. Adipose Gene Expression Prior to Weight Loss Can Differentiate and Weakly Predict Dietary Responders. PloS One (2007) 2(12):e1344. doi: 10.1371/journal.pone.0001344 18094752 PMC2147074

[7] Hirsch J Batchelor B. Adipose Tissue Cellularity in Human Obesity. Clin Endocrinol Metab (1976) 5(2):299–311. doi: 10.1016/s0300-595x(76)80023-0 1085232

[8] Bjorntorp P. Number and Size of Adipose Tissue Fat Cells in Relation to Metabolism in Human Obesity. Metabolism (1971) 20(7):703–13. doi: 10.1016/0026-0495(71)90084-9 5090134

[9] Salans LB Cushman SW Weismann RE. Studies of Human Adipose Tissue. Adipose Cell Size and Number in Nonobese and Obese Patients. J Clin Invest (1973) 52(4):929–41. doi: 10.1172/JCI107258 PMC302341 4693656

[10] Stenkula KG Erlanson-Albertsson C. Adipose Cell Size: Importance in Health and Disease. Am J Physiol Regul Integr Comp Physiol (2018) 315(2):R284–R95. doi: 10.1152/ajpregu.00257.2017 29641234

[11] Yang J Eliasson B Smith U Cushman SW Sherman AS. The Size of Large Adipose Cells Is a Predictor of Insulin Resistance in First-Degree Relatives of Type 2 Diabetic Patients. Obes (Silver Spring) (2012) 20(5):932–8. doi: 10.1038/oby.2011.371 PMC3457700 22240722

[12] Ktotkiewski M Sjostrom L Bjorntorp P Smith U. Regional Adipose Tissue Cellularity in Relation to Metabolism in Young and Middle-Aged Women. Metabolism (1975) 24(6):703–10. doi: 10.1016/0026-0495(75)90038-4 1128235

[13] Roberts R Hodson L Dennis AL Neville MJ Humphreys SM Harnden KE. Markers of *De Novo* Lipogenesis in Adipose Tissue: Associations With Small Adipocytes and Insulin Sensitivity in Humans. Diabetologia (2009) 52(5):882–90. doi: 10.1007/s00125-009-1300-4 19252892

[14] Lundgren M Svensson M Lindmark S Renstrom F Ruge T Eriksson JW. Fat Cell Enlargement Is an Independent Marker of Insulin Resistance and 'Hyperleptinaemia'. Diabetologia (2007) 50(3):625–33. doi: 10.1007/s00125-006-0572-1 17216279

[15] Kloting N Fasshauer M Dietrich A Kovacs P Schon MR Kern M. Insulin-Sensitive Obesity. Am J Physiol Endocrinol Metab (2010) 299(3):E506–15. doi: 10.1152/ajpendo.00586.2009 20570822

[16] Imbeault P Lemieux S Prud'homme D Tremblay A Nadeau A Despres JP. Relationship of Visceral Adipose Tissue to Metabolic Risk Factors for Coronary Heart Disease: Is There a Contribution of Subcutaneous Fat Cell Hypertrophy? Metabolism (1999) 48(3):355–62. doi: 10.1016/s0026-0495(99)90085-9 10094113

[17] Heinonen S Saarinen L Naukkarinen J Rodriguez A Fruhbeck G Hakkarainen A. Adipocyte Morphology and Implications for Metabolic Derangements in Acquired Obesity. Int J Obes (Lond) (2014) 38(11):1423–31. doi: 10.1038/ijo.2014.31 24549139

[18] Veilleux A Caron-Jobin M Noel S Laberge PY Tchernof A. Visceral Adipocyte Hypertrophy Is Associated With Dyslipidemia Independent of Body Composition and Fat Distribution in Women. Diabetes (2011) 60(5):1504–11. doi: 10.2337/db10-1039 PMC3292324 21421806

[19] Hoffstedt J Arner E Wahrenberg H Andersson DP Qvisth V Lofgren P. Regional Impact of Adipose Tissue Morphology on the Metabolic Profile in Morbid Obesity. Diabetologia (2010) 53(12):2496–503. doi: 10.1007/s00125-010-1889-3 20830466

[20] Cotillard A Poitou C Torcivia A Bouillot JL Dietrich A Kloting N. Adipocyte Size Threshold Matters: Link With Risk of Type 2 Diabetes and Improved Insulin Resistance After Gastric Bypass. J Clin Endocrinol Metab (2014) 99(8):E1466–70. doi: 10.1210/jc.2014-1074 24780048

[21] Petaja EM Sevastianova K Hakkarainen A Orho-Melander M Lundbom N Yki-Jarvinen H. Adipocyte Size Is Associated With Nafld Independent of Obesity, Fat Distribution, and Pnpla3 Genotype. Obes (Silver Spring) (2013) 21(6):1174–9. doi: 10.1002/oby.20114 23913731

[22] Wree A Schlattjan M Bechmann LP Claudel T Sowa JP Stojakovic T. Adipocyte Cell Size, Free Fatty Acids and Apolipoproteins Are Associated With Non-Alcoholic Liver Injury Progression in Severely Obese Patients. Metabolism (2014) 63(12):1542–52. doi: 10.1016/j.metabol.2014.09.001 25267016

[23] Naryzhnaya NV Koshelskaya OA Kologrivova IV Kharitonova OA Evtushenko VV Boshchenko AA. Hypertrophy and Insulin Resistance of Epicardial Adipose Tissue Adipocytes: Association With the Coronary Artery Disease Severity. Biomedicines (2021) 9(1):64. doi: 10.3390/biomedicines9010064 33440802 PMC7827040

[24] Vianello E Dozio E Arnaboldi F Marazzi MG Martinelli C Lamont J. Epicardial Adipocyte Hypertrophy: Association With M1-Polarization and Toll-Like Receptor Pathways in Coronary Artery Disease Patients. Nutr Metab Cardiovasc Dis (2016) 26(3):246–53. doi: 10.1016/j.numecd.2015.12.005 26841679

[25] Manneras-Holm L Leonhardt H Kullberg J Jennische E Oden A Holm G. Adipose Tissue Has Aberrant Morphology and Function in Pcos: Enlarged Adipocytes and Low Serum Adiponectin, But Not Circulating Sex Steroids, Are Strongly Associated With Insulin Resistance. J Clin Endocrinol Metab (2011) 96(2):E304–11. doi: 10.1210/jc.2010-1290 21084397

[26] Salans LB Knittle JL Hirsch J. The Role of Adipose Cell Size and Adipose Tissue Insulin Sensitivity in the Carbohydrate Intolerance of Human Obesity. J Clin Invest (1968) 47(1):153–65. doi: 10.1172/JCI105705 PMC297156 16695937

[27] Andersson DP Eriksson Hogling D Thorell A Toft E Qvisth V Naslund E. Changes in Subcutaneous Fat Cell Volume and Insulin Sensitivity After Weight Loss. Diabetes Care (2014) 37(7):1831–6. doi: 10.2337/dc13-2395 24760260

[28] Goossens GH Moors CC van der Zijl NJ Venteclef N Alili R Jocken JW. Valsartan Improves Adipose Tissue Function in Humans With Impaired Glucose Metabolism: A Randomized Placebo-Controlled Double-Blind Trial. PloS One (2012) 7(6):e39930. doi: 10.1371/journal.pone.0039930 22768174 PMC3386933

[29] Rizkalla SW Prifti E Cotillard A Pelloux V Rouault C Allouche R. Differential Effects of Macronutrient Content in 2 Energy-Restricted Diets on Cardiovascular Risk Factors and Adipose Tissue Cell Size in Moderately Obese Individuals: A Randomized Controlled Trial. Am J Clin Nutr (2012) 95(1):49–63. doi: 10.3945/ajcn.111.017277 22170375

[30] McLaughlin T Abbasi F Lamendola C Yee G Carter S Cushman SW. Dietary Weight Loss in Insulin-Resistant Non-Obese Humans: Metabolic Benefits and Relationship to Adipose Cell Size. Nutr Metab Cardiovasc Dis (2019) 29(1):62–8. doi: 10.1016/j.numecd.2018.09.014 PMC6410738 30497926

[31] Skurk T Alberti-Huber C Herder C Hauner H. Relationship Between Adipocyte Size and Adipokine Expression and Secretion. J Clin Endocrinol Metab (2007) 92(3):1023–33. doi: 10.1210/jc.2006-1055 17164304

[32] Bahceci M Gokalp D Bahceci S Tuzcu A Atmaca S Arikan S. The Correlation Between Adiposity and Adiponectin, Tumor Necrosis Factor Alpha, Interleukin-6 and High Sensitivity C-Reactive Protein Levels. Is Adipocyte Size Associated With Inflammation in Adults? J Endocrinol Invest (2007) 30(3):210–4. doi: 10.1007/BF03347427 17505154

[33] Monteiro R de Castro PM Calhau C Azevedo I. Adipocyte Size and Liability to Cell Death. Obes Surg (2006) 16(6):804–6. doi: 10.1381/096089206777346600 16756747

[34] Li Q Hagberg CE Silva Cascales H Lang S Hyvonen MT Salehzadeh F. Obesity and Hyperinsulinemia Drive Adipocytes to Activate a Cell Cycle Program and Senesce. Nat Med (2021) 27(11):1941–53. doi: 10.1038/s41591-021-01501-8 34608330

[35] Michaud A Boulet MM Veilleux A Noel S Paris G Tchernof A. Abdominal Subcutaneous and Omental Adipocyte Morphology and Its Relation to Gene Expression, Lipolysis and Adipocytokine Levels in Women. Metabolism (2014) 63(3):372–81. doi: 10.1016/j.metabol.2013.11.007 24369916

[36] Laurencikiene J Skurk T Kulyte A Heden P Astrom G Sjolin E. Regulation of Lipolysis in Small and Large Fat Cells of the Same Subject. J Clin Endocrinol Metab (2011) 96(12):E2045–9. doi: 10.1210/jc.2011-1702 21994963

[37] Farnier C Krief S Blache M Diot-Dupuy F Mory G Ferre P. Adipocyte Functions Are Modulated by Cell Size Change: Potential Involvement of an Integrin/Erk Signalling Pathway. Int J Obes Relat Metab Disord (2003) 27(10):1178–86. doi: 10.1038/sj.ijo.0802399 14513065

[38] Weyer C Foley JE Bogardus C Tataranni PA Pratley RE. Enlarged Subcutaneous Abdominal Adipocyte Size, But Not Obesity Itself, Predicts Type Ii Diabetes Independent of Insulin Resistance. Diabetologia (2000) 43(12):1498–506. doi: 10.1007/s001250051560 11151758

[39] Lonn M Mehlig K Bengtsson C Lissner L. Adipocyte Size Predicts Incidence of Type 2 Diabetes in Women. FASEB J (2010) 24(1):326–31. doi: 10.1096/fj.09-133058 19741173

[40] Johannsen DL Tchoukalova Y Tam CS Covington JD Xie W Schwarz JM. Effect of 8 Weeks of Overfeeding on Ectopic Fat Deposition and Insulin Sensitivity: Testing the "Adipose Tissue Expandability" Hypothesis. Diabetes Care (2014) 37(10):2789–97. doi: 10.2337/dc14-0761 PMC4170127 25011943

[41] McLaughlin T Craig C Liu LF Perelman D Allister C Spielman D. Adipose Cell Size and Regional Fat Deposition as Predictors of Metabolic Response to Overfeeding in Insulin-Resistant and Insulin-Sensitive Humans. Diabetes (2016) 65(5):1245–54. doi: 10.2337/db15-1213 PMC5384627 26884438

[42] Melchor-Lopez A Suarez-Cuenca JA Banderas-Lares DZ Pena-Sosa G Salamanca-Garcia M Vera-Gomez E. Identification of Adipose Tissue-Related Predictors of the Reduction in Cardiovascular Risk Induced by Metabolic Surgery. J Int Med Res (2021) 49(5):3000605211012569. doi: 10.1177/03000605211012569 34024182 PMC8150427

[43] Eriksson-Hogling D Andersson DP Backdahl J Hoffstedt J Rossner S Thorell A. Adipose Tissue Morphology Predicts Improved Insulin Sensitivity Following Moderate or Pronounced Weight Loss. Int J Obes (Lond) (2015) 39(6):893–8. doi: 10.1038/ijo.2015.18 25666530

[44] Laforest S Labrecque J Michaud A Cianflone K Tchernof A. Adipocyte Size as a Determinant of Metabolic Disease and Adipose Tissue Dysfunction. Crit Rev Clin Lab Sci (2015) 52(6):301–13. doi: 10.3109/10408363.2015.1041582 26292076

[45] Mundi MS Karpyak MV Koutsari C Votruba SB O'Brien PC Jensen MD. Body Fat Distribution, Adipocyte Size, and Metabolic Characteristics of Nondiabetic Adults. J Clin Endocrinol Metab (2010) 95(1):67–73. doi: 10.1210/jc.2009-1353 19890025 PMC2805479

[46] Ledoux S Coupaye M Essig M Msika S Roy C Queguiner I. Traditional Anthropometric Parameters Still Predict Metabolic Disorders in Women With Severe Obesity. Obes (Silver Spring) (2010) 18(5):1026–32. doi: 10.1038/oby.2009.349 19851304

[47] Meena VP Seenu V Sharma MC Mallick SR Bhalla AS Gupta N. Relationship of Adipocyte Size With Adiposity and Metabolic Risk Factors in Asian Indians. PloS One (2014) 9(9):e108421. doi: 10.1371/journal.pone.0108421 25251402 PMC4177391

[48] Brook CG Lloyd JK. Adipose Cell Size and Glucose Tolerance in Obese Children and Effects of Diet. Arch Dis Child (1973) 48(4):301–4. doi: 10.1136/adc.48.4.301 PMC1648325 4705934

[49] Larson-Meyer DE Heilbronn LK Redman LM Newcomer BR Frisard MI Anton S. Effect of Calorie Restriction With or Without Exercise on Insulin Sensitivity, Beta-Cell Function, Fat Cell Size, and Ectopic Lipid in Overweight Subjects. Diabetes Care (2006) 29(6):1337–44. doi: 10.2337/dc05-2565 PMC2677812 16732018

[50] Koenen TB Tack CJ Kroese JM Hermus AR Sweep FC van der Laak J. Pioglitazone Treatment Enlarges Subcutaneous Adipocytes in Insulin-Resistant Patients. J Clin Endocrinol Metab (2009) 94(11):4453–7. doi: 10.1210/jc.2009-0517 19820024

[51] Boden G. Obesity, Insulin Resistance and Free Fatty Acids. Curr Opin Endocrinol Diabetes Obes (2011) 18(2):139–43. doi: 10.1097/MED.0b013e3283444b09 PMC3169796 21297467

[52] Laforest S Michaud A Paris G Pelletier M Vidal H Geloen A. Comparative Analysis of Three Human Adipocyte Size Measurement Methods and Their Relevance for Cardiometabolic Risk. Obes (Silver Spring) (2017) 25(1):122–31. doi: 10.1002/oby.21697 27883275

[53] Golan R Shelef I Rudich A Gepner Y Shemesh E Chassidim Y. Abdominal Superficial Subcutaneous Fat: A Putative Distinct Protective Fat Subdepot in Type 2 Diabetes. Diabetes Care (2012) 35(3):640–7. doi: 10.2337/dc11-1583 PMC3322677 22344612

[54] DeBari MK Abbott RD. Adipose Tissue Fibrosis: Mechanisms, Models, and Importance. Int J Mol Sci (2020) 21(17):6030. doi: 10.3390/ijms21176030 PMC7503256 32825788

[55] Fried SK. Adipocyte Size Redux. Obes (Silver Spring) (2017) 25(1):15. doi: 10.1002/oby.21717 PMC5182077 27935267

[56] Arner E Westermark PO Spalding KL Britton T Ryden M Frisen J. Adipocyte Turnover: Relevance to Human Adipose Tissue Morphology. Diabetes (2010) 59(1):105–9. doi: 10.2337/db09-0942 PMC2797910 19846802

[57] McLaughlin T Sherman A Tsao P Gonzalez O Yee G Lamendola C. Enhanced Proportion of Small Adipose Cells in Insulin-Resistant Vs Insulin-Sensitive Obese Individuals Implicates Impaired Adipogenesis. Diabetologia (2007) 50(8):1707–15. doi: 10.1007/s00125-007-0708-y 17549449

[58] Kursawe R Eszlinger M Narayan D Liu T Bazuine M Cali AM. Cellularity and Adipogenic Profile of the Abdominal Subcutaneous Adipose Tissue From Obese Adolescents: Association With Insulin Resistance and Hepatic Steatosis. Diabetes (2010) 59(9):2288–96. doi: 10.2337/db10-0113 PMC2927952 20805387

[59] Hotamisligil GS Arner P Caro JF Atkinson RL Spiegelman BM. Increased Adipose Tissue Expression of Tumor Necrosis Factor-Alpha in Human Obesity and Insulin Resistance. J Clin Invest (1995) 95(5):2409–15. doi: 10.1172/JCI117936 PMC295872 7738205

[60] Weisberg SP McCann D Desai M Rosenbaum M Leibel RL Ferrante AW Jr. Obesity Is Associated With Macrophage Accumulation in Adipose Tissue. J Clin Invest (2003) 112(12):1796–808. doi: 10.1172/JCI19246 PMC296995 14679176

[61] Xu H Barnes GT Yang Q Tan G Yang D Chou CJ. Chronic Inflammation in Fat Plays a Crucial Role in the Development of Obesity-Related Insulin Resistance. J Clin Invest (2003) 112(12):1821–30. doi: 10.1172/JCI19451 PMC296998 14679177

[62] Reilly SM Saltiel AR. Adapting to Obesity With Adipose Tissue Inflammation. Nat Rev Endocrinol (2017) 13(11):633–43. doi: 10.1038/nrendo.2017.90 28799554

[63] Kawai T Autieri MV Scalia R. Adipose Tissue Inflammation and Metabolic Dysfunction in Obesity. Am J Physiol Cell Physiol (2021) 320(3):C375–C91. doi: 10.1152/ajpcell.00379.2020 PMC8294624 33356944

[64] Bluher M. Adipose Tissue Inflammation: A Cause or Consequence of Obesity-Related Insulin Resistance? Clin Sci (Lond) (2016) 130(18):1603–14. doi: 10.1042/CS20160005 27503945

[65] Cancello R Tordjman J Poitou C Guilhem G Bouillot JL Hugol D. Increased Infiltration of Macrophages in Omental Adipose Tissue Is Associated With Marked Hepatic Lesions in Morbid Human Obesity. Diabetes (2006) 55(6):1554–61. doi: 10.2337/db06-0133 16731817

[66] Tordjman J Poitou C Hugol D Bouillot JL Basdevant A Bedossa P. Association Between Omental Adipose Tissue Macrophages and Liver Histopathology in Morbid Obesity: Influence of Glycemic Status. J Hepatol (2009) 51(2):354–62. doi: 10.1016/j.jhep.2009.02.031 19464069

[67] Harman-Boehm I Bluher M Redel H Sion-Vardy N Ovadia S Avinoach E. Macrophage Infiltration Into Omental Versus Subcutaneous Fat Across Different Populations: Effect of Regional Adiposity and the Comorbidities of Obesity. J Clin Endocrinol Metab (2007) 92(6):2240–7. doi: 10.1210/jc.2006-1811 17374712

[68] Hardy OT Perugini RA Nicoloro SM Gallagher-Dorval K Puri V Straubhaar J. Body Mass Index-Independent Inflammation in Omental Adipose Tissue Associated With Insulin Resistance in Morbid Obesity. Surg Obes Relat Dis (2011) 7(1):60–7. doi: 10.1016/j.soard.2010.05.013 PMC2980798 20678967

[69] van Beek L Lips MA Visser A Pijl H Ioan-Facsinay A Toes R. Increased Systemic and Adipose Tissue Inflammation Differentiates Obese Women With T2dm From Obese Women With Normal Glucose Tolerance. Metabolism (2014) 63(4):492–501. doi: 10.1016/j.metabol.2013.12.002 24467914

[70] Goldstein N Kezerle Y Gepner Y Haim Y Pecht T Gazit R. Higher Mast Cell Accumulation in Human Adipose Tissues Defines Clinically Favorable Obesity Sub-Phenotypes. Cells (2020) 9(6):1508. doi: 10.3390/cells9061508 PMC7349306 32575785

[71] Lopez-Perez D Redruello-Romero A Garcia-Rubio J Arana C Garcia-Escudero LA Tamayo F. In Patients With Obesity, the Number of Adipose Tissue Mast Cells Is Significantly Lower in Subjects With Type 2 Diabetes. Front Immunol (2021) 12:664576. doi: 10.3389/fimmu.2021.664576 34093556 PMC8177010

[72] Divoux A Moutel S Poitou C Lacasa D Veyrie N Aissat A. Mast Cells in Human Adipose Tissue: Link With Morbid Obesity, Inflammatory Status, and Diabetes. J Clin Endocrinol Metab (2012) 97(9):E1677–85. doi: 10.1210/jc.2012-1532 22745246

[73] Bertola A Ciucci T Rousseau D Bourlier V Duffaut C Bonnafous S. Identification of Adipose Tissue Dendritic Cells Correlated With Obesity-Associated Insulin-Resistance and Inducing Th17 Responses in Mice and Patients. Diabetes (2012) 61(9):2238–47. doi: 10.2337/db11-1274 PMC3425417 22596049

[74] McLaughlin T Liu LF Lamendola C Shen L Morton J Rivas H. T-Cell Profile in Adipose Tissue Is Associated With Insulin Resistance and Systemic Inflammation in Humans. Arterioscler Thromb Vasc Biol (2014) 34(12):2637–43. doi: 10.1161/ATVBAHA.114.304636 PMC4445971 25341798

[75] Maixner N Pecht T Haim Y Chalifa-Caspi V Goldstein N Tarnovscki T. A Trail-Tl1a Paracrine Network Involving Adipocytes, Macrophages, and Lymphocytes Induces Adipose Tissue Dysfunction Downstream of E2f1 in Human Obesity. Diabetes (2020) 69(11):2310–23. doi: 10.2337/db19-1231 PMC7576574 32732304

[76] Cancello R Henegar C Viguerie N Taleb S Poitou C Rouault C. Reduction of Macrophage Infiltration and Chemoattractant Gene Expression Changes in White Adipose Tissue of Morbidly Obese Subjects After Surgery-Induced Weight Loss. Diabetes (2005) 54(8):2277–86. doi: 10.2337/diabetes.54.8.2277 16046292

[77] Bradley D Conte C Mittendorfer B Eagon JC Varela JE Fabbrini E. Gastric Bypass and Banding Equally Improve Insulin Sensitivity and Beta Cell Function. J Clin Invest (2012) 122(12):4667–74. doi: 10.1172/JCI64895 PMC3512168 23187122

[78] Hagman DK Larson I Kuzma JN Cromer G Makar K Rubinow KB. The Short-Term and Long-Term Effects of Bariatric/Metabolic Surgery on Subcutaneous Adipose Tissue Inflammation in Humans. Metabolism (2017) 70:12–22. doi: 10.1016/j.metabol.2017.01.030 28403936 PMC5407411

[79] Kratz M Hagman DK Kuzma JN Foster-Schubert KE Chan CP Stewart S. Improvements in Glycemic Control After Gastric Bypass Occur Despite Persistent Adipose Tissue Inflammation. Obes (Silver Spring) (2016) 24(7):1438–45. doi: 10.1002/oby.21524 PMC4925247 27228052

[80] Magkos F Fraterrigo G Yoshino J Luecking C Kirbach K Kelly SC. Effects of Moderate and Subsequent Progressive Weight Loss on Metabolic Function and Adipose Tissue Biology in Humans With Obesity. Cell Metab (2016) 23(4):591–601. doi: 10.1016/j.cmet.2016.02.005 26916363 PMC4833627

[81] Boden G Homko C Barrero CA Stein TP Chen X Cheung P. Excessive Caloric Intake Acutely Causes Oxidative Stress, Glut4 Carbonylation, and Insulin Resistance in Healthy Men. Sci Transl Med (2015) 7(304):304re7. doi: 10.1126/scitranslmed.aac4765 PMC5600191 26355033

[82] Burhans MS Hagman DK Kuzma JN Schmidt KA Kratz M. Contribution of Adipose Tissue Inflammation to the Development of Type 2 Diabetes Mellitus. Compr Physiol (2018) 9(1):1–58. doi: 10.1002/cphy.c170040 30549014 PMC6557583

[83] Berg AH Scherer PE. Adipose Tissue, Inflammation, and Cardiovascular Disease. Circ Res (2005) 96(9):939–49. doi: 10.1161/01.RES.0000163635.62927.34 15890981

[84] Labrecque J Laforest S Michaud A Biertho L Tchernof A. Impact of Bariatric Surgery on White Adipose Tissue Inflammation. Can J Diabetes (2017) 41(4):407–17. doi: 10.1016/j.jcjd.2016.12.003 28365202

[85] Trachta P Dostalova I Haluzikova D Kasalicky M Kavalkova P Drapalova J. Laparoscopic Sleeve Gastrectomy Ameliorates Mrna Expression of Inflammation-Related Genes in Subcutaneous Adipose Tissue But Not in Peripheral Monocytes of Obese Patients. Mol Cell Endocrinol (2014) 383(1-2):96–102. doi: 10.1016/j.mce.2013.11.013 24291610

[86] Donath MY Meier DT Boni-Schnetzler M. Inflammation in the Pathophysiology and Therapy of Cardiometabolic Disease. Endocr Rev (2019) 40(4):1080–91. doi: 10.1210/er.2019-00002 PMC6624792 31127805

[87] Goldfine AB Shoelson SE. Therapeutic Approaches Targeting Inflammation for Diabetes and Associated Cardiovascular Risk. J Clin Invest (2017) 127(1):83–93. doi: 10.1172/JCI88884 28045401 PMC5199685

[88] Mariman EC Wang P. Adipocyte Extracellular Matrix Composition, Dynamics and Role in Obesity. Cell Mol Life Sci (2010) 67(8):1277–92. doi: 10.1007/s00018-010-0263-4 PMC2839497 20107860

[89] Marcelin G Gautier EL Clément K. Adipose Tissue Fibrosis in Obesity: Etiology and Challenges. Annu Rev Physiol (2021) 84:135–55. doi: 10.1146/annurev-physiol-060721-092930 34752708

[90] Pasarica M Gowronska-Kozak B Burk D Remedios I Hymel D Gimble J. Adipose Tissue Collagen Vi in Obesity. J Clin Endocrinol Metab (2009) 94(12):5155–62. doi: 10.1210/jc.2009-0947 PMC2819828 19837927

[91] Dankel SN Svärd J Matthä S Claussnitzer M Klöting N Glunk V. Col6a3 Expression in Adipocytes Associates With Insulin Resistance and Depends on Pparγ and Adipocyte Size. Obes (Silver Spring) (2014) 22(8):1807–13. doi: 10.1002/oby.20758 24719315

[92] Yoshino J Patterson BW Klein S. Adipose Tissue Ctgf Expression Is Associated With Adiposity and Insulin Resistance in Humans. Obes (Silver Spring) (2019) 27(6):957–62. doi: 10.1002/oby.22463 PMC6533148 31004409

[93] Henninger AM Eliasson B Jenndahl LE Hammarstedt A. Adipocyte Hypertrophy, Inflammation and Fibrosis Characterize Subcutaneous Adipose Tissue of Healthy, Non-Obese Subjects Predisposed to Type 2 Diabetes. PloS One (2014) 9(8):e105262. doi: 10.1371/journal.pone.0105262 25148116 PMC4141784

[94] Chabot K Gauthier MS Garneau PY Rabasa-Lhoret R. Evolution of Subcutaneous Adipose Tissue Fibrosis After Bariatric Surgery. Diabetes Metab (2017) 43(2):125–33. doi: 10.1016/j.diabet.2016.10.004 27843076

[95] Guglielmi V Cardellini M Cinti F Corgosinho F Cardolini I D'Adamo M. Omental Adipose Tissue Fibrosis and Insulin Resistance in Severe Obesity. Nutr Diabetes (2015) 5(8):e175. doi: 10.1038/nutd.2015.22 26258766 PMC4558556

[96] Leven AS Gieseler RK Schlattjan M Schreiter T Niedergethmann M Baars T. Association of Cell Death Mechanisms and Fibrosis in Visceral White Adipose Tissue With Pathological Alterations in the Liver of Morbidly Obese Patients With Nafld. Adipocyte (2021) 10(1):558–73. doi: 10.1080/21623945.2021.1982164 PMC8583086 34743657

[97] Abdennour M Reggio S Le Naour G Liu Y Poitou C Aron-Wisnewsky J. Association of Adipose Tissue and Liver Fibrosis With Tissue Stiffness in Morbid Obesity: Links With Diabetes and Bmi Loss After Gastric Bypass. J Clin Endocrinol Metab (2014) 99(3):898–907. doi: 10.1210/jc.2013-3253 24423338

[98] Divoux A Tordjman J Lacasa D Veyrie N Hugol D Aissat A. Fibrosis in Human Adipose Tissue: Composition, Distribution, and Link With Lipid Metabolism and Fat Mass Loss. Diabetes (2010) 59(11):2817–25. doi: 10.2337/db10-0585 PMC2963540 20713683

[99] Muir LA Neeley CK Meyer KA Baker NA Brosius AM Washabaugh AR. Adipose Tissue Fibrosis, Hypertrophy, and Hyperplasia: Correlations With Diabetes in Human Obesity. Obes (Silver Spring) (2016) 24(3):597–605. doi: 10.1002/oby.21377 PMC4920141 26916240

[100] Henegar C Tordjman J Achard V Lacasa D Cremer I Guerre-Millo M. Adipose Tissue Transcriptomic Signature Highlights the Pathological Relevance of Extracellular Matrix in Human Obesity. Genome Biol (2008) 9(1):R14. doi: 10.1186/gb-2008-9-1-r14 18208606 PMC2395253

[101] Cancello R Zulian A Gentilini D Mencarelli M Della Barba A Maffei M. Permanence of Molecular Features of Obesity in Subcutaneous Adipose Tissue of Ex-Obese Subjects. Int J Obes (Lond) (2013) 37(6):867–73. doi: 10.1038/ijo.2013.7 23399771

[102] Buechler C Krautbauer S Eisinger K. Adipose Tissue Fibrosis. World J Diabetes (2015) 6(4):548–53. doi: 10.4239/wjd.v6.i4.548 PMC4434075 25987952

[103] Hardy OT Czech MP Corvera S. What Causes the Insulin Resistance Underlying Obesity? Curr Opin Endocrinol Diabetes Obes (2012) 19(2):81–7. doi: 10.1097/MED.0b013e3283514e13 PMC4038351 22327367

[104] Pasarica M Sereda OR Redman LM Albarado DC Hymel DT Roan LE. Reduced Adipose Tissue Oxygenation in Human Obesity: Evidence for Rarefaction, Macrophage Chemotaxis, and Inflammation Without an Angiogenic Response. Diabetes (2009) 58(3):718–25. doi: 10.2337/db08-1098 PMC2646071 19074987

[105] Bel Lassen P Charlotte F Liu Y Bedossa P Le Naour G Tordjman J. The Fat Score, a Fibrosis Score of Adipose Tissue: Predicting Weight-Loss Outcome After Gastric Bypass. J Clin Endocrinol Metab (2017) 102(7):2443–53. doi: 10.1210/jc.2017-00138 28419237

[106] Spencer M Unal R Zhu B Rasouli N McGehee RE Jr. Peterson CA. Adipose Tissue Extracellular Matrix and Vascular Abnormalities in Obesity and Insulin Resistance. J Clin Endocrinol Metab (2011) 96(12):E1990–8. doi: 10.1210/jc.2011-1567 PMC3232606 21994960

[107] Vila Isabelle K Badin P-M Marques M-A Monbrun L Lefort C Mir L. Immune Cell Toll-Like Receptor 4 Mediates the Development of Obesity- and Endotoxemia-Associated Adipose Tissue Fibrosis. Cell Rep (2014) 7(4):1116–29. doi: 10.1016/j.celrep.2014.03.062 24794440

[108] Liu Y Aron-Wisnewsky J Marcelin G Genser L Le Naour G Torcivia A. Accumulation and Changes in Composition of Collagens in Subcutaneous Adipose Tissue After Bariatric Surgery. J Clin Endocrinol Metab (2016) 101(1):293–304. doi: 10.1210/jc.2015-3348 26583585

[109] Mutch DM Tordjman J Pelloux V Hanczar B Henegar C Poitou C. Needle and Surgical Biopsy Techniques Differentially Affect Adipose Tissue Gene Expression Profiles. Am J Clin Nutr (2009) 89(1):51–7. doi: 10.3945/ajcn.2008.26802 19056587

[110] Sasso M Abdennour M Liu Y Hazrak H Aron-Wisnewsky J Bouillot J-L. Relevance of Adipose Tissue Stiffness Evaluated by Transient Elastography (Adiposcan™) in Morbidly Obese Patients Before Bariatric Surgery. Phys Proc (2015) 70:1264–8. doi: 10.1016/j.phpro.2015.08.281

[111] Bouazizi K Zarai M Marquet F Aron-Wisnewsky J Clément K Redheuil A. Adipose Tissue Fibrosis Assessed by High Resolution Ex Vivo Mri as a Hallmark of Tissue Alteration in Morbid Obesity. Quant Imaging Med Surg (2021) 11(5):2162–8. doi: 10.21037/qims-20-879 PMC8047361 33936996

[112] Alba DL Farooq JA Lin MYC Schafer AL Shepherd J Koliwad SK. Subcutaneous Fat Fibrosis Links Obesity to Insulin Resistance in Chinese Americans. J Clin Endocrinol Metab (2018) 103(9):3194–204. doi: 10.1210/jc.2017-02301 PMC6126891 29846621

[113] Munoz A Abate N Chandalia M. Adipose Tissue Collagen and Inflammation in Nonobese Asian Indian Men. J Clin Endocrinol Metab (2013) 98(8):E1360–3. doi: 10.1210/jc.2012-3841 PMC3733857 23780376

[114] Von Bank H Kirsh C Simcox J. Aging Adipose: Depot Location Dictates Age-Associated Expansion and Dysfunction. Ageing Res Rev (2021) 67:101259. doi: 10.1016/j.arr.2021.101259 33515751 PMC8379680

[115] Cooper TC Simmons EB Webb K Burns JL Kushner RF. Trends in Weight Regain Following Roux-En-Y Gastric Bypass (Rygb) Bariatric Surgery. Obes Surg (2015) 25(8):1474–81. doi: 10.1007/s11695-014-1560-z 25595383

[116] Aron-Wisnewsky J Sokolovska N Liu Y Comaneshter DS Vinker S Pecht T. The Advanced-Diarem Score Improves Prediction of Diabetes Remission 1 Year Post-Roux-En-Y Gastric Bypass. Diabetologia (2017) 60(10):1892–902. doi: 10.1007/s00125-017-4371-7 28733906

[117] Marquez-Quinones A Mutch DM Debard C Wang P Combes M Roussel B. Adipose Tissue Transcriptome Reflects Variations Between Subjects With Continued Weight Loss and Subjects Regaining Weight 6 Mo After Caloric Restriction Independent of Energy Intake. Am J Clin Nutr (2010) 92(4):975–84. doi: 10.3945/ajcn.2010.29808 20739421

[118] Giardina S Hernandez-Alonso P Salas-Salvado J Rabassa-Soler A Bullo M. Modulation of Human Subcutaneous Adipose Tissue Microrna Profile Associated With Changes in Adiposity-Related Parameters. Mol Nutr Food Res (2018) 62(2):1700594. doi: 10.1002/mnfr.201700594 29024341

[119] Vijay J Gauthier MF Biswell RL Louiselle DA Johnston JJ Cheung WA. Single-Cell Analysis of Human Adipose Tissue Identifies Depot and Disease Specific Cell Types. Nat Metab (2020) 2(1):97–109. doi: 10.1038/s42255-019-0152-6 32066997 PMC7025882

[120] Pecht T Gutman-Tirosh A Bashan N Rudich A. Peripheral Blood Leucocyte Subclasses as Potential Biomarkers of Adipose Tissue Inflammation and Obesity Subphenotypes in Humans. Obes Rev (2014) 15(4):322–37. doi: 10.1111/obr.12133 24251825

[121] Pecht T Haim Y Bashan N Shapiro H Harman-Boehm I Kirshtein B. Circulating Blood Monocyte Subclasses and Lipid-Laden Adipose Tissue Macrophages in Human Obesity. PloS One (2016) 11(7):e0159350. doi: 10.1371/journal.pone.0159350 27442250 PMC4956051

[122] Shapiro H Pecht T Shaco-Levy R Harman-Boehm I Kirshtein B Kuperman Y. Adipose Tissue Foam Cells Are Present in Human Obesity. J Clin Endocrinol Metab (2013) 98(3):1173–81. doi: 10.1210/jc.2012-2745 23372170

[123] Henninger J Eliasson B Smith U Rawshani A. Identification of Markers That Distinguish Adipose Tissue and Glucose and Insulin Metabolism Using a Multi-Modal Machine Learning Approach. Sci Rep (2021) 11(1):17050. doi: 10.1038/s41598-021-95688-y 34426590 PMC8382765

[124] Sattar N Wannamethee G Sarwar N Tchernova J Cherry L Wallace AM. Adiponectin and Coronary Heart Disease: A Prospective Study and Meta-Analysis. Circulation (2006) 114(7):623–9. doi: 10.1161/CIRCULATIONAHA.106.618918 16894037

[125] Lindberg S Jensen JS Bjerre M Frystyk J Flyvbjerg A Jeppesen J. Low Adiponectin Levels at Baseline and Decreasing Adiponectin Levels Over 10 Years of Follow-Up Predict Risk of the Metabolic Syndrome. Diabetes Metab (2017) 43(2):134–9. doi: 10.1016/j.diabet.2016.07.027 27639310

[126] Kim JY Ahn SV Yoon JH Koh SB Yoon J Yoo BS. Prospective Study of Serum Adiponectin and Incident Metabolic Syndrome: The Arirang Study. Diabetes Care (2013) 36(6):1547–53. doi: 10.2337/dc12-0223 PMC3661834 23275369

